# Emergence of multi drug resistant nonfermentative Acinetobacter spp in a tertiary care hospital

**DOI:** 10.1186/1471-2334-12-S1-P28

**Published:** 2012-05-04

**Authors:** Shyamkuwar Amol, Deogade Narendra, Wadher Bharat, Roychoudhury Kunal

**Affiliations:** 1 Post Graduate Teaching Department of Microbiology, RTM Nagpur University, Nagpur-440033, India; 2 Department of Microbiology, Indira Gandhi Government Medical College, Nagpur-440018, India; 3 PG Department of Microbiology, Seth Kesarimal Porwal College, Kamptee-441002, India

## Background

*Acinetobacter* are gram negative, catalase positive, oxidase negative, nonmotile, non fermenting cocobacilli. The emergence of *Acinetobacter* infection is uncommon in organ systems that have a high fluid content. The nosocomial infections including, respiratory tract, CSF, peritoneal fluid, urinary tract, and endotracheal aspirtes (ET) are the place where they have the capabilities to accumulate and cause in-hospital and in-community infections. The rate of emergence of *Acinetobacter* spp with multi drug resistance property is increasing in different geographical regions. Due to the properties of MDR the recent treatments of such infections has become difficult. In the present study evaluation of emergence of MDR nonfermentative *Acinetobacter* spp was done from the niche of organ system of patients with high fluid content.

## Method

The nonfermentative *Acinetobacter* spp were isolated from different clinical samples according to standard procedures and Gilardi schemes. The antibiotyping of Acinetobacter was done by disk diffusion method as per CLSI standards.

## Results

31.12% (62/193) of nonfermentative *Acinetobacter* spp were isolated from different clinical samples. The ratio of resistance against different antibiotics among Female:Male was found to be 1:1.38. All *Acinetobacter* spp were multi-resistant and showed different multi drug resistance pattern. *Acinetobacter* spp from samples showed varied resistance including, 60% resistant strain from wound swabs, 50% from Pus, 28-30% from ET aspirates and blood.

## Conclusion

*Acinetobacter* spp posing significant problem worldwide and increasingly responsible for numerous infections. Our study shows the emergence of high rate of MDR of *Acinetobacter* spp against 18 antibiotics belonging to different groups.

